# Dissecting the MAPK signaling landscape in malignant melanoma: from BRAF and NRAS mutations to precision combination therapies

**DOI:** 10.3389/fcell.2025.1723066

**Published:** 2026-01-14

**Authors:** Xiaobo Fang, Shuangyin Wang, Shuangxing Fu

**Affiliations:** 1 Affiliated Hospital of Shaoxing University, Shaoxing, Zhejiang, China; 2 The Affiliated Hospital of Hangzhou Normal University, Hangzhou, Zhejiang, China

**Keywords:** BRAF inhibitors, combination therapy, MAPK pathway, melanoma, NRAS mutations, precision medicine

## Abstract

Malignant melanoma is an aggressive skin malignancy with a complex molecular landscape and limited treatment durability in advanced stages. Aberrations in the MAPK pathway—most notably BRAF and NRAS mutations—have catalyzed the development of targeted therapies, particularly BRAF/MEK inhibitors, which have transformed outcomes in BRAF-mutant melanoma. However, resistance remains prevalent, driven by MAPK reactivation, epigenetic rewiring, and tumor microenvironmental feedback. In NRAS-mutant subtypes, MEK inhibition, CDK4/6 blockade, and immune checkpoint inhibition offer partial efficacy, yet monotherapies fail to achieve sustained responses. Emerging strategies focus on combinatorial regimens targeting RAF-MEK-ERK and PI3K-AKT axes, alongside immunotherapeutic integration. Rarer alterations in KIT and RTKs also define actionable subsets. This review synthesizes recent mechanistic insights and therapeutic advances in mutation-driven melanoma, highlighting the promise of biomarker-guided combination strategies and signaling crosstalk disruption as the next frontier in precision oncology.

## 1 Introduction

Malignant melanoma is the most lethal form of skin cancer, accounting for 80% of skin cancer–related deaths, largely due to its strong metastatic propensity and complex molecular heterogeneity (Force et al., 2025). Although surgical resection is often curative in early-stage disease, advanced melanoma remains difficult to treat, as conventional systemic regimens typically deliver low objective response rates (ORRs) and substantial toxicity (Davis et al., 2019; Smalley et al., 2016; Kim et al., 2013). The discovery of oncogenic alterations within the mitogen-activated protein kinase (MAPK) pathway, particularly BRAF mutations, has reshaped therapeutic paradigms (Reddi et al., 2022). Accordingly, targeted strategies, including BRAF inhibitors combined with MEK inhibitors such as trametinib or cobimetinib, have produced clinically meaningful benefits in patients with BRAF-mutant melanoma (Eroglu and Ribas, 2016; Eroglu and Ozgun, 2018).

Nevertheless, durability of response is frequently curtailed by resistance mechanisms, including MAPK pathway reactivation and intratumoral heterogeneity, necessitating integrated treatment modalities. Dual blockade of BRAF and MEK, as well as immunotherapy combinations involving PD-1/PD-L1 inhibitors, have been explored to mitigate resistance and enhance therapeutic efficacy (Haugh et al., 2021; Ribas et al., 2019; Ferrucci et al., 2020). In NRAS-mutant melanoma, MEK inhibition and immune checkpoint blockade represent pivotal approaches, though clinical benefit varies across demographic groups (Dummer et al., 2017; Lebbé et al., 2020). Additionally, rarer genetic alterations, such as those in KIT, GNAQ, GNA11, and NF1, are implicated in MAPK hyperactivation, particularly in BRAF- and NRAS-wild-type subsets (Guhan et al., 2021; Cosgarea et al., 2017). These variants not only shape melanoma progression but also contribute to treatment resistance and are increasingly employed as biomarkers to inform precision interventions (Revythis et al., 2021). Combinatorial strategies that integrate targeted pathway suppression with immunomodulation are under active development, aiming to circumvent resistance and restrain metastasis (Kim and Kim, 2024). This review delineates current advancements and persisting obstacles in the pursuit of personalized melanoma therapy.

## 2 BRAF/MAPK signaling pathway in malignant melanoma

### 2.1 The MAPK signaling pathway and therapeutic targets

Aberrant activation of the MAPK cascade is tightly linked to melanoma initiation and proliferation and may further promote metastatic competence through downstream effectors (Wellbrock and Arozarena, 2016). Signaling is triggered by RTK dimerization, RAS activation via GRB2/SOS, and stepwise phosphorylation along the RAF–MEK–ERK axis (Favre, 2014; Zhang et al., 2022; Savoia et al., 2019). Nuclear ERK1/2 phosphorylates transcription factors (ELK1) and induces proto-oncogenic programs, including c-Fos and cyclin D1. BRAF mutations drive constitutive MEK/ERK signaling, providing a strong rationale for targeted therapy (Maik-Rachline et al., 2019; Arkenau et al., 2011). Clinically, BRAF inhibitors (vemurafenib, dabrafenib) and MEK inhibition (trametinib) outperform chemotherapy (Chapman et al., 2017). However, resistance commonly arises through NRAS activation and MAPK pathway reactivation via multiple routes, including RTK upregulation (EGFR, PDGFRβ), BRAF splice variants, MEK1/2 mutations, MAP3K8 (COT) overexpression, and NF1 loss (Klein et al., 2008). Epigenetic silencing of DUSP and NF1, together with miRNA-mediated suppression of ERK inhibitors (SPRY, SPRED), can further amplify MAPK output (Ablain et al., 2021; Masliah-Planchon et al., 2016). Although the causal link between MAPK hyperactivation and metastasis remains under active investigation, ERK-dependent induction of MMP-2/-9, Rho/ROCK-driven cytoskeletal remodeling, and VEGF-A–mediated angiogenesis have been mechanistically associated with enhanced invasion. Accordingly, emerging strategies emphasize combination regimens that co-target BRAF/MEK and complementary pathways (imatinib) to counter resistance and reshape the tumor microenvironment (Aseervatham, 2020; Rager et al., 2022).

### 2.2 BRAF kinase alterations and targeted therapy in malignant melanoma

The RAF kinase family (ARAF, BRAF, and RAF1) constitutes a central axis of the MAPK pathway, with BRAF acting as a principal oncogenic driver due to its frequent mutational activation (Zhang et al., 2023; Braicu et al., 2019). Notably, the V600E mutation enables MEK phosphorylation independent of RAS signaling, triggering sustained ERK pathway activity (Tian and Guo, 2020). The V600E substitution disrupts autoinhibitory control at the ATP-binding site, enabling constitutive MEK phosphorylation independent of upstream RAS input and sustaining ERK-driven tumorigenesis via cyclin D1 upregulation and CDK4 activation (Hirai et al., 2021). Intriguingly, BRAF mutations are less prevalent in East Asian populations, a disparity not fully explained by ultraviolet exposure, as BRAF V600E is not a UV signature mutation (Ren et al., 2022; Mao et al., 2021; Wang et al., 2022). This difference likely reflects the higher proportion of acral and mucosal melanomas in these populations—subtypes typically exhibiting lower BRAF mutation rates and relative enrichment of KIT and NRAS alterations—along with ethnic-specific germline variation and distinct tumor mutational burdens (Ren et al., 2022; Hida et al., 2024). Mechanistically, the V600E substitution disrupts BRAF autoinhibition within the ATP-binding region, leading to constitutive ERK pathway activation that promotes proliferation and apoptosis resistance, thereby providing a strong rationale for selective BRAF inhibition (Maloney et al., 2021). Despite the clinical activity of selective BRAF inhibitors (vemurafenib and dabrafenib), paradoxical ERK activation remains a major limitation. Whereas BRAF^V600E signals largely as a RAS-independent monomer, wild-type BRAF and RAF1 (CRAF) typically require RAS-driven dimerization for activation (Poulikakos et al., 2010; Girotti et al., 2015; Cox and Der, 2012). In RAS-activated settings, BRAF inhibitors bind one RAF protomer within a dimer but allosterically hinder engagement of the second protomer, generating a hemi-bound dimer that paradoxically enhances MEK/ERK signaling (Sievert et al., 2013; Vasta et al., 2023). This mechanism is implicated in characteristic cutaneous toxicities, including keratoacanthomas and cutaneous squamous cell carcinomas (Adelmann et al., 2016; Chu et al., 2012). Accordingly, MEK co-inhibition has become a cornerstone of BRAF-targeted therapy by suppressing ERK reactivation, improving tolerability and therapeutic durability (Eroglu and Ribas, 2016; Kakadia et al., 2018; McArthur, 2015). This combination strategy exemplifies the necessity of mechanistic insight in guiding rational therapeutic design and minimizing adverse events in BRAF-mutant melanoma.

### 2.3 MEK inhibitors for malignant melanoma

MEK1/2, dual-specificity kinases that phosphorylate ERK1/2 at the conserved Thr-Glu-Tyr motif, represent a central node in MAPK signaling (Cargnello and Roux, 2011; Barbosa et al., 2021). BRAF V600E-mutant melanomas are typically MEK-dependent and thus sensitive to MEK inhibition, whereas NRAS/KRAS-driven tumors frequently bypass this dependency through compensatory PI3K/AKT activation, limiting monotherapy efficacy (Kakadia et al., 2018; Luebker and Koepsell, 2019; Posch et al., 2013). Allosteric MEK inhibitors reconfigure the ATP-binding cleft, suppressing ERK phosphorylation, inducing G1/S arrest, and initiating mitochondrial apoptosis. BRAF V600E cell lines exhibit reduced IC50 values versus wild-type, reinforcing their clinical translatability (Solit et al., 2006; Gonzalez-Del Pino et al., 2021). First-generation MEK inhibitors (CI-1040) established druggability but were constrained by poor stability (Montagut and Settleman, 2009); the second-generation agent PD0325901 improved potency (including Val211 engagement) yet was curtailed by dose-limiting toxicity (Boasberg et al., 2011). These culminated in trametinib, a third-generation FDA-approved MEK1/2 inhibitor that blocks ERK activation, induces G1 arrest, and suppresses tumor growth *in vivo* (Gilmartin et al., 2011). In BRAF inhibitor-naïve patients, trametinib showed superior response rates (33%) relative to pretreated (17%) or BRAF wild-type (10%) cohorts (Infante et al., 2012; Falchook et al., 2012). Nonetheless, resistance—via ERK reactivation, PI3K/AKT/mTOR signaling, and immunosuppressive remodeling—has driven combination strategies, including pairing with PD-1 blockade (Liu et al., 2025; Zhong et al., 2022; Deken et al., 2016). Second-generation MEK inhibitors, such as selumetinib (AZD6244), suppress ERK1/2 phosphorylation by stabilizing an inactive MEK conformation through interaction with the αC-helix. Although they show activity in BRAF-mutant tumors, combinations with docetaxel can yield genotype-dependent outcomes, with synergy in BRAF-mutant settings but heightened toxicity in NRAS-mutant cells (Catalanotti et al., 2013; Patel et al., 2013; Gupta et al., 2014; Kim and Patel, 2014). Cobimetinib, another FDA-approved MEK1/2 inhibitor, reduces paradoxical ERK reactivation when combined with vemurafenib (Ascierto et al., 2021; Abdel-Wahab et al., 2014). These findings highlight the value of vertical MAPK blockade and underscore the necessity for mutation-guided combinations in melanoma treatment.

### 2.4 Imatinib mesylate targets KIT pathway

Imatinib mesylate marked a pivotal advancement in precision oncology, especially for melanomas driven by KIT mutations (Jung et al., 2022). As the inaugural KIT/PDGFRA tyrosine kinase inhibitor approved by the FDA (Zhou et al., 2024), imatinib acts by obstructing the ATP-binding domain (residues Asp810–Val814–Val817), thereby disrupting pathological KIT signaling. Its efficacy is notably superior in tumors harboring KIT exon 11 mutations, where it shows enhanced selectivity over the wild-type counterpart (Guo et al., 2011). Importantly, imatinib is not the only KIT inhibitor evaluated in melanoma; second-generation agents, including nilotinib, dasatinib, and sunitinib, have shown clinical activity across multiple KIT mutation subtypes and resistance contexts (Pham et al., 2020; Steeb et al., 2021; Kaveti et al., 2025; Larkin et al., 2024; Guo et al., 2017). Nilotinib and sunitinib exhibit greater potency against selected imatinib-resistant variants, whereas dasatinib provides broader coverage through multi-kinase inhibition (Kalinsky et al., 2017; Serrano et al., 2019). These agents are being assessed as monotherapies and in combination regimens to improve outcomes in KIT-driven melanoma (Pham et al., 2020; Kaveti et al., 2025). However, accumulating evidence suggests that KIT inhibition alone is frequently undermined by pathway redundancy and escape signaling. Consequently, rational combinations—particularly dual KIT and MEK inhibition—are gaining traction, with early clinical evaluation underway (NCT04598009) to deepen MAPK suppression and curb compensatory reactivation.

## 3 Rationale and paradigm of targeted combination therapy

### 3.1 BRAF and MEK inhibitor combination

Although BRAF inhibitors have substantially improved outcomes in BRAF-mutant melanoma, their clinical benefit is constrained by dose-limiting toxicities, limited response durability, and the near-universal emergence of acquired resistance, motivating combination-based strategies (Zhong et al., 2022; Liu et al., 2017). A dominant resistance mechanism is MAPK pathway reactivation via multiple molecular adaptations, including EGFR upregulation with RTK-mediated feedback–driven ERK reactivation (Saei and Eichhorn, 2019; Ji et al., 2021); paradoxical ERK activation that enables outgrowth of RAS-mutant subclones; epigenetic NF1 inactivation with impaired RAS-GTPase function; expression of BRAF p61 splice isoforms that promote RAF autophosphorylation; activating alterations in MEK1/2 or MLK1–4 that support bypass signaling (Tse and Verkhivker, 2016; Bartnik et al., 2019; Czarnecka et al., 2020); and immune editing associated with altered cytokine outputs and increased CD8^+^ T-cell infiltration (Haas et al., 2021; Grecea-Balaj et al., 2025). Mechanistically, resistance is frequently maintained by RAF homodimerization that preserves MAPK signaling under drug pressure and by aberrant ERK feedback loops that reconstitute oncogenic signaling within the tumor microenvironment (Sievert et al., 2013; Meierjohann, 2017). Dual blockade more effectively suppresses ERK phosphorylation at its activation residues (Thr202/Tyr204), thereby enhancing apoptosis and delaying resistance (McArthur, 2015; Su et al., 2012). In line with this model, genomic profiling of resistant populations suggests a synthetic-lethal vulnerability characterized by heightened sensitivity to MEK inhibition, consistent with clinical observations that combination therapy prolongs median PFS and reduces resistance incidence (Abdel-Rahman et al., 2016).

### 3.2 BRAF and MEK inhibitor in clinical

The Phase III coBRIM trial (NCT01689519) demonstrated that vemurafenib plus cobimetinib significantly outperformed vemurafenib monotherapy in BRAF V600–mutant melanoma, with improved efficacy and a manageable safety profile—76% of grade 3–4 toxicities occurred early in treatment, and MEK-mediated ERK suppression led to a 60% reduction in acute kidney injury incidence (Ascierto et al., 2016; Teuma et al., 2017). The COMBI-d study similarly confirmed the efficacy of dabrafenib plus trametinib, enhancing PFS and ORR (Long et al., 2014). Mechanistically, trametinib inhibits MEK1/2 and dampens ERK reactivation, while dabrafenib induces BIM and suppresses cyclin D1, though febrile adverse events were more frequent (Robert et al., 2016). Current melanoma treatment strategies prioritize rational combinations to enhance pharmacodynamic synergy while minimizing toxicity (Smalley et al., 2016). These include modulation of BRAF/MEK dosing to reduce severe toxicities, vertical co-targeting of bypass nodes such as PI3Kβ/δ or AKT (NCT01941927), and horizontal integration with PD-1 blockade (NCT02967692) (Luke et al., 2017). While encorafenib and binimetinib, second-generation BRAF and MEK inhibitors, have been approved for BRAF V600E/K-mutant melanoma, they are not indicated for NRAS- or wild-type tumors (Baik et al., 2024). According to Schadendorf (2025), their use remains confined to select genomic subsets, and broad application should be approached with caution. These findings underscore the necessity of genomic stratification to guide therapy selection and maximize clinical benefit.

## 4 Molecular mechanisms and signal transduction features of NRAS-driven oncogenic networks in malignant melanoma

**FIGURE 1:**
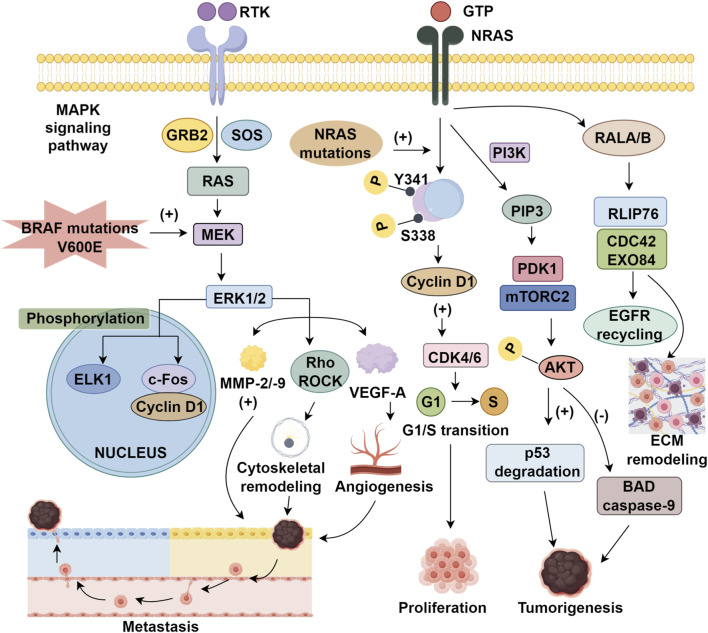
The MAPK signaling pathway and therapeutic targets in melanoma.

The RAS gene family—NRAS, KRAS, and HRAS—encodes small GTPases that function as binary molecular switches, cycling between GTP-bound active and GDP-bound inactive states, regulated by GEFs and GAPs (Tsai et al., 2015; Hobbs et al., 2016). In malignant melanoma, NRAS mutations—most frequently at codon 61—result in constitutive GTP-loading, driving sustained oncogenic signaling and conferring a more aggressive phenotype than BRAF-mutant or wild-type melanomas (Lally et al., 2022; Zablocka et al., 2023). NRAS orchestrates tumorigenesis through three principal effector pathways. First, MAPK cascade activation via RAF1 (formerly CRAF) homodimers phosphorylated at S338/Y341 promotes cyclin D1 upregulation and CDK4/6 activation, facilitating G1/S transition (Wang P. et al., 2023; Wee and Wang, 2017). Second, PI3K–AKT–mTOR signaling is amplified through PIP3-mediated recruitment of PDK1 and mTORC2, leading to AKT phosphorylation, suppression of apoptotic mediators (BAD, caspase-9), p53 degradation, and enhanced translational machinery (Peng et al., 2022). Third, RAL GTPase activation stimulates EGFR recycling and extracellular matrix (ECM) remodeling via RLIP76 and CDC42/EXO84, promoting invasiveness (Zipfel et al., 2010; Falsetti et al., 2007) (Figure 1). This multifaceted signaling confers intrinsic resistance to BRAF inhibitors and limits MEK inhibitor efficacy (Phadke and Smalley, 2023). Accordingly, therapeutic strategies must address network redundancy and crosstalk. Although MEK inhibitors like binimetinib and pimasertib have demonstrated only modest activity, with limited progression-free survival (PFS) and no overall survival (OS) advantage (Dummer et al., 2017), combinatorial regimens offer a promising alternative. Notably, MEK–CDK4/6 co-inhibition has improved ORR in Phase II trials, delaying resistance emergence (Lebbé et al., 2020). Given these limitations, PD-1 immune checkpoint inhibitors have been endorsed by NCCN and ESMO as frontline therapies for NRAS-mutant melanoma (Michielin et al., 2019; Swetter et al., 2024). Yet, interregional variability complicates clinical generalizability. European cohorts report ORRs 28% higher in NRAS-mutant tumors versus wild-type (Jaeger et al., 2023), whereas Asian studies reveal attenuated responses across cutaneous and extracutaneous subtypes (Zhou et al., 2021). These disparities underscore the importance of molecular and ethnic context in therapeutic design. Collectively, NRAS-mutant melanoma exemplifies the challenges of targeting non-ligandable oncoproteins and the need for precision oncology paradigms integrating immune modulation with multi-node pathway inhibition, tailored to distinct genetic architectures.

## 5 Potential targets focusing on RAS pathway

### 5.1 Monotherapy and FTIs in NRAS-mutant melanoma

Efforts to develop targeted therapies for melanoma harboring NRAS mutations encounter significant molecular impediments. In contrast to the structurally defined drug-binding pocket observed in BRAF V600 alterations, the canonical NRAS mutation sites (such as Q61R/L) present a major obstacle due to the absence of suitable ligandable conformations within their nucleotide-binding regions (Phadke and Smalley, 2023; Muñoz-Couselo et al., 2017). The GTP-binding cleft is characterized by restrictive geometry and exceptionally high-affinity interactions at the picomolar scale, which collectively render conventional competitive inhibition strategies largely futile (Mattox et al., 2019). Moreover, approaches attempting to directly antagonize GTP itself have consistently yielded negative outcomes (Ryan and Corcoran, 2018). Historically, research emphasis shifted toward interfering with RAS-associated post-translational modifications. For instance, farnesyltransferase inhibitors were designed to hinder the membrane anchoring of p21RAS by blocking farnesylation at its C-terminal cysteine residue. Nevertheless, clinical trials evaluating this strategy produced disappointing outcomes; in a Phase II cohort involving 14 patients with NRAS-mutant melanoma, no objective therapeutic responses were observed, resulting in early discontinuation of the trial and highlighting the divergence between mechanistic rationale and actual clinical benefit (Gajewski et al., 2012). Contemporary investigations have broadened toward cross-target strategies exemplified by sotorasib, an allosteric KRAS G12C inhibitor that stabilizes KRAS in its inactive state and has demonstrated substantial clinical activity across multiple solid tumors. Although its utility in NRAS G12C–mutant melanoma remains speculative, the mechanistic rationale warrants systematic clinical evaluation to define its translational potential (Hong et al., 2020).

### 5.2 RAF-MAPK and ERK pathway inhibitors

Given the intrinsic challenges of directly targeting NRAS—stemming from its high-affinity GTP binding and limited druggable pockets—therapeutic development in NRAS-mutant melanoma has largely pivoted to downstream inhibition within the RAF–MAPK cascade (Wang et al., 2025; Fernandez et al., 2023). Preclinical work indicates that dual suppression of BRAF and RAF1 can significantly restrain tumor growth, providing a mechanistic rationale for pan-RAF inhibition (Karoulia et al., 2016; Vakana et al., 2017). Clinically, belvarafenib (RG6185/HM95573) has shown only modest activity in early-phase studies (Yen et al., 2021). By contrast, the MEK1/2 inhibitor tunlametinib (HL-085) has reported more favorable outcomes, achieving a 35% objective response rate (ORR) that increased to 39% in patients refractory to prior immunotherapies, and outperforming binimetinib in cross-study comparisons (Wang X. et al., 2023). Targeting ERK1/2, the terminal effectors of MAPK signaling, provides an alternative axis. Although preclinical ERK inhibitors such as VX-11e can suppress feedback-driven pathway reactivation (Adam et al., 2020), clinical activity has thus far been limited, with ulixertinib achieving a 13.5% ORR (Mendzelevski et al., 2018). Consequently, combination approaches co-targeting MEK and ERK are being pursued to blunt adaptive resistance and improve response durability (Adam et al., 2020). Beyond vertical pathway blockade, pan-RAS(ON) inhibitors represent a conceptual advance. In contrast to earlier strategies that failed clinically (farnesyltransferase inhibition), emerging agents such as RMC-7977 and RMC-6236 are designed to engage the switch II pocket of GTP-loaded RAS, thereby interrupting oncogenic signaling at its source (Holderfield et al., 2024; Drosten and Barbacid, 2025). RMC-7977 has been reported to induce tumor regression in NRAS- and KRAS-mutant preclinical models, including melanoma xenografts (Anastacio Da Costa Carvalho et al., 2025). RMC-6236 is undergoing clinical evaluation (NCT05379985) and has shown potent MAPK suppression with early partial responses in heavily pretreated patients (Filis et al., 2025). By targeting active RAS, these agents may mitigate the feedback reactivation that often undermines MEK-directed therapy and could offer improved therapeutic durability. As clinical development matures, pan-RAS(ON) inhibitors may reshape treatment paradigms for NRAS-driven melanoma (Supplementary Table S1).

### 5.3 Novel combination strategies for NRAS-mutant melanoma

Monotherapies have shown limited efficacy in NRAS-mutant melanoma due to complex downstream signaling networks and innate resistance mechanisms. While pan-RAF inhibitors are constrained by feedback-mediated MAPK reactivation, their combination with trametinib has yielded improved outcomes, as evidenced by a 40% objective response rate (ORR) in trial NCT03284502 and a 46.7% ORR with naporafenib plus trametinib (Atefi et al., 2015; de Braud et al., 2023). In contrast, dual inhibition strategies targeting the PI3K/AKT axis have produced inconsistent results. Alpelisib plus binimetinib achieved 20% ORR (Bartnik et al., 2019), whereas trametinib with GSK2141795 failed to elicit clinical responses in a Phase I study (NCT01941927) (Algazi et al., 2018), highlighting the limitations of non-stratified combination therapies. Conversely, biomarker-guided approaches have demonstrated enhanced efficacy. NRAS-mutant tumors with CDKN2A, CDK4, or CCND1 alterations exhibited increased sensitivity to ribociclib combined with binimetinib, underscoring the relevance of precision medicine (Kinsey et al., 2025). Parallel strategies incorporating autophagy inhibition have gained traction; clinical trial NCT03979651 is evaluating hydroxychloroquine as an adjunct to MEK inhibition to overcome resistance (Mehnert et al., 2022). Additionally, molecular profiling reveals overexpression of RTKs—particularly Axl, ERBB2, c-MET, and EGFR—in NRAS-driven melanoma, implicating these receptors in drug resistance (Sabbah et al., 2021; Robinson et al., 2017). Accordingly, RTK-targeted combinations such as sorafenib and tivantinib have shown superior efficacy in NRAS-mutant tumors relative to wild-type controls (Rager et al., 2022; Puzanov et al., 2015; Fedorenko et al., 2013). These findings reinforce the necessity of genetically informed, multi-targeted strategies to address adaptive resistance and optimize therapeutic outcomes in this biologically complex melanoma subtype.

## 6 Conclusion

Despite significant advances in targeted and immunotherapy, malignant melanoma continues to present substantial clinical challenges, particularly in treatment-resistant and NRAS-mutant subtypes. Monotherapies targeting MAPK signaling, including BRAF and MEK inhibitors, have provided transient benefits but are undermined by adaptive resistance mechanisms such as pathway reactivation, compensatory signaling loops, and immunosuppressive remodeling. In NRAS-mutant melanoma, the therapeutic landscape is further complicated by the lack of direct RAS inhibitors and the limited efficacy of MEK blockade alone. Combination therapies—integrating MEK inhibitors with CDK4/6 inhibitors, autophagy modulators, or immune checkpoint inhibitors—have emerged as promising avenues, especially in genetically defined subgroups. Moreover, the identification of co-occurring alterations in PI3K/AKT, RTKs, and cell-cycle regulators has refined patient stratification and enabled rational design of biomarker-driven regimens. The therapeutic efficacy of KIT inhibitors in specific molecular contexts underscores the broader potential of precision-guided interventions. Moving forward, multi-targeted combination strategies that concurrently disrupt oncogenic signaling and reshape the tumor immune microenvironment are poised to redefine melanoma treatment paradigms. Continued integration of genomic, epigenetic, and immunologic profiling will be essential to overcome resistance and extend durable responses in this biologically diverse malignancy.
